# Utilization of Stimuli-Responsive Biomaterials in the Formulation of Cancer Vaccines

**DOI:** 10.3390/jfb14050247

**Published:** 2023-04-28

**Authors:** Arun Kumar Singh, Rishabha Malviya, Bhupendra Prajapati, Sudarshan Singh, Priyanshi Goyal

**Affiliations:** 1 Department of Pharmacy, School of Medical and Allied Sciences, Galgotias University, Greater Noida 203201, India; arunthakur01996@gmail.com (A.K.S.); priyanshi.21smas2020003@galgotiasuniversity.edu.in (P.G.); 2 Shree S. K. Patel College of Pharmaceutical Education and Research, Ganpat University, Kherva 384012, India; 3 Department of Pharmaceutical Sciences, Faculty of Pharmacy, Chiang Mai University, Chiang Mai 50200, Thailand

**Keywords:** vaccine, cancer, biomaterial, stimuli-responsive, polymer, patient care

## Abstract

Immunology research has focused on developing cancer vaccines to increase the number of tumor-specific effector cells and their ability to fight cancer over the last few decades. There is a lack of professional success in vaccines compared to checkpoint blockade and adoptive T-cell treatment. The vaccine’s inadequate delivery method and antigen selection are most likely to blame for the poor results. Antigen-specific vaccines have recently shown promising results in preclinical and early clinical investigations. To target particular cells and trigger the best immune response possible against malignancies, it is necessary to design a highly efficient and secure delivery method for cancer vaccines; however, enormous challenges must be overcome. Current research is focused on developing stimulus-responsive biomaterials, which are a subset of the range of levels of materials, to enhance therapeutic efficacy and safety and better regulate the transport and distribution of cancer immunotherapy in vivo. A concise analysis of current developments in the area of biomaterials that respond to stimuli has been provided in brief research. Current and anticipated future challenges and opportunities in the sector are also highlighted.

## 1. Introduction

Cancer vaccination as an immunotherapy to increase the immune system’s anti-tumor immunity has been extensively studied in recent decades [1]. There have been several successful vaccines for infectious illnesses, but no vaccine has yet been created for cancer. There are just a few vaccines now available for virus-related diseases, such as cervical cancer linked to the human papillomavirus. The cancer vaccine Provenge^®^ has been licensed and approved for the delivery of anticancer drugs [2]. Some therapeutic cancer vaccines had mixed results in clinical trials so far. At this time, cancer vaccines are unsuccessful because it is difficult to find a tumor-specific antigen and because vaccine components cannot be managed at the tissue and cell levels. Antigen selection may have been overcome by recent breakthroughs in neoantigen-based cancer vaccines, allowing for more powerful cancer immunizations. It is still challenging to develop an effective and safe delivery method for immunotherapy targeting neoantigens [3,4]. Drug delivery systems based on biomaterials have made tremendous strides during the last several decades. From synthetic polymers to biomolecules to inorganic crystals, a vast range of biological materials are available in various configurations and sizes. When it comes to biomaterials, all four sciences have a hand in it [5]. Because of this newer technology, a wide range of complex and different physiological needs may be met by various biomaterials [6,7,8]. To tackle vaccine delivery difficulties, immunologists and nanomaterials scientists have turned to biomaterials. Nanoparticle-based cancer vaccines have shown considerable promise in preclinical and clinical research, but human trials have not yet been conducted [9,10,11]. Peer-reviewed papers provide a comprehensive description of these initiatives. One of the most effective drug delivery systems is a carrier is “smart” materials with precision stimuli-responsiveness [12]. A broad variety of physiological extracellular matrix stimuli, including pH, redox potential, and temperature, may be responded to by stimuli-sensitive biomaterials to distribute and release drugs and vaccine components [13]. The release of external stimuli such as light or ultrasound may also be controlled using these biomaterials. This accuracy in time and place, made achievable by a whole new class of biomaterials, has made it feasible to administer medications with greater effectiveness and less “off-target” damage. Researchers have extensively covered the subject of stimuli-responsive drug delivery devices in several good review articles [14,15]. To date, no systematic study has been conducted on stimuli-responsive materials for the administration of cancer vaccines. Non-cellular subunit cancer vaccine-sensitive delivery is the subject of this research. There are both advantages and downsides to using stimuli-sensitive biomaterials in subunit cancer vaccines. This sector has both obstacles and possibilities in the years to come, as examined. This review article focuses on the utilization of stimuli-responsive biomaterials in the formulation of cancer vaccines.

## 2. Designing an Effective Cancer Vaccine

**Figure 1:**
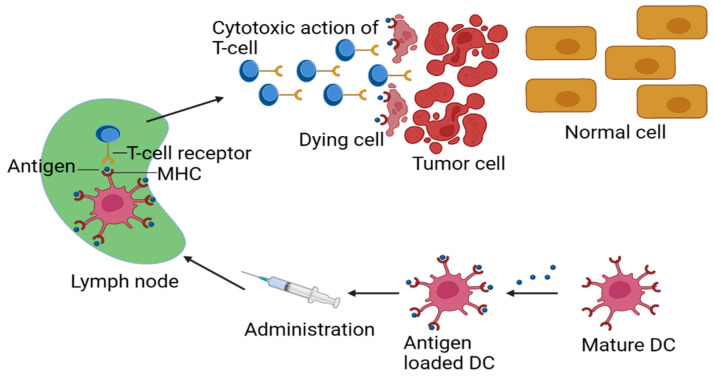
Schematic representation of designing principles for an effective cancer vaccine MHC (Major Histocompatibility Complex), DC (dendritic cell).

Experts have lauded an effective cancer vaccine [16,17]. Vaccine administration to non-cellular cancer cells and general design issues are discussed in the following section. For an effective immune response, vaccines must activate the adaptive immune system, which comprises B and T cells. B cells create anti-allergy antibodies as part of a humoral reaction to an allergen. CD4+ cells, for example, aid in the activation of other immune cells, but CD8+ CTLs are critical to the “cellular immunological response”, which is the process of cell killing [18]. An anticancer vaccination induces a significant CD8+ T cell response that effectively kills cancer cells [17,19]. To process and present antigens, two stages follow endocytic endocytosis in the immunization process: to make antigen-specific epitopes that bind to MHC (Major Histocompatibility Complex) molecules, proteases process endosomal/lysosomal antigens released into the cytosol. This is the typical approach for subunit vaccinations. MHC Class II molecules deliver endosomal antigens degraded by proteases to CD4+ helper T cells, as shown in Figure 1. For an immune response to be triggered, CD8+ T cells must cross-present. T-cell immunity is heavily reliant on good cross-presentation for a strong cancer vaccination [20].

## 3. Using Stimuli-Responsive Biomaterials to Manage the Distribution of the Cancer Vaccine

An antigen produced by the tumor or associated tissues and an adjuvant that enhances an immune response are two of the most common components of cancer vaccines. The subunit antigen of the cancer vaccine must be given an adjuvant to increase its immunogenicity. Pattern-recognition receptor agonist compounds are increasingly common in modern molecular additives such as Toll-like receptor (TLR) agonist chemicals [21,22]. For a successful cancer vaccination, the antigen and auxiliary must be delivered to the APCs (Antigen-Presenting Cells) and lymphoid tissues.

To make a cancer vaccine successful, two important delivery issues must be addressed simultaneously: (1) directing immune system components from vaccines as well as APCs and (2) improving the cross-presentation of antigens. Deliveries at the cellular or tissue level are seen in both examples. Intravenous cancer vaccines should target lymphoid cells, such as lymph nodes or mucosa-associated lymphoid tissues, to maximize antigen presentation efficacy (MALTs). These organs, which have higher concentrations of DCs (dendritic cells) than peripheral tissues, may be primed by APCs to produce naive T cells. Soluble antigens and molecular adjuvants are too tiny to be effective, and the risk of systemic toxicity is too great, making immunization ineffective. Additional challenges that must be overcome to eradicate cancer cells include the acquisition of vaccines by APCs and the nearly exclusive loading of MHC Class II molecules from subunit vaccinations. These challenges must be overcome to trigger an effective anticancer immune response; cancer vaccines must be administered on a platform that is both effective and adaptable.

Biomaterials that respond to stimuli have been employed to solve the delivery problem of cancer vaccines. To manage the processing of immune system antigens, biomaterials with adaptable chemical designs are used.

As part of this discussion, we look at some of the most recent developments made in cancer vaccines that use responsive biomaterials and the possible drawbacks of this technique (Table 1). There are two kinds of responsive biomaterials: those that enhance LN (lymph node) and APC targeting as well as strategies that improve cross-presentation based on their activities

## 4. Responsive Biomaterials Improvement of LN and APC Targeting

Many biomaterials have been created to trigger anticancer CD8+ T cell responses to drive vaccine components toward lymph nodes (LNs). NPs (Nanoparticles) may flow directly to LNs in the 9–100 nm range, while APCs are required to collect and transport bigger NPs [23,24]. When it comes to delivering vaccine ingredients into lymph nodes, particle biomaterials must be optimized [22]. To improve internalization by expert APCs such as DCs, NP-based delivery methods are coated with DC-targeting compounds such as anti-CD11c antibodies and mannose [25,26]. The delivery of antigens and adjuvants to LNs and APCs may be improved by combining nanoparticles of optimal size and surface modifications [9,27]. Additionally, researchers are focusing on biomaterial responsiveness in addition to size and surface modification to better target LNs and internalize through APCs. For example, Lynn et al. [28,29,30,31,32,33,34,35] have shown self-assembling vaccine–polymer combinations. A coil–peptide–antigen-fusion protein heterodimer was created by adding peptides to the In P (NIPAM-co (Ma-Ahx TT)) conjugated to Toll-like receptor 7/8 agonists. The thermosensitive dimer that maintained solubility at normal temperature was injected into mice, resulting in immunogenic particles. A more precise chemical structure, superior stability, and enhanced LN targeting are advantages of soluble antigens against particulates, but more APC internalization is an advantage of particulates [36,37,38,39,40]. This was a one-of-a-kind method of delivery. Resistance to the non-responsive vaccine was much higher in BALB/c mice treated with thermoresponsive immunization [41,42,43,44,45]. Despite the widespread use of thermoresponsive biomaterials in the delivery of anticancer drugs, few efforts have been made to administer cancer vaccines using this technology [46,47]. This research shows that thermal self-assembly may improve LN targeting. Cancer vaccines may be delivered directly to lymph nodes (LNs) via a variety of methods [48,49,50]. However, there are just a few attempts at developing biomaterials that may help cancer vaccines target lymph nodes. Similarly, responsive biomaterials might be used to deliver cancer vaccines to lymph nodes, since these materials have highly programmable features.

## 5. pH-Responsive Vaccine Delivery System

Extracellular and intracellular pH measurements reveal that the endocytic spaces of DCs have a somewhat acidic pH. While being surrounded by endosomal structures, proteases are more quickly destroyed as a consequence of antigen import by endocytosis and the consequent acidification of the lysosome [51].

Low endosomal pH is a suitable internal signal for pH-responsive vaccine delivery techniques for regulating antigen production [52]. Reacting to changes in pH, this intracellular distribution may be achieved by using acid-catalyzed disintegration, particle phase shift, and the “proton sponge effect”. When it comes to the administration of cancer vaccines, pH-responsive biomaterials have attracted the greatest attention.

### 5.1. Acid-Labile Biomaterials with the Ability to Change pH

Making pH-responsive cancer vaccines is simple, as acid-labile polymeric NPs are used to encapsulate vaccine components. Certain molecular linkers are stable at neutral or slightly basic pH, but a rapid breakdown in acidic cellular compartments forms the foundation of this kind of vaccine carrier.

A rapid increase in the number of antigen molecules disturbs the endosomal membrane, enabling antigens released from ruptured endosomes to be released more easily. Polymeric NPs for immunization delivery were pioneered by the Fréchet group and others [53,54]. Making use of microgel linkers that cross-linked polymer chains to create an acid-labile ketal-derived moiety, they created a vaccine delivery system that adjusts to the surrounding pH level.

Polyacrylamide and degrading polyurethanes may be used to create a variety of cross-linked biomaterials [29,55]. pH-responsive vaccination significantly boosted the CD8+ T cell cross-priming and effector function in mice versus vaccines that were not sensitive to pH changes or that were less responsive. A DC-targeting antibody may be added to particle vaccinations to boost their ability to target DCs [56,57].

The first-time antigenic proteins were delivered to DCs, they were not accompanied by adjuvants. When DCs were exposed to an acid-labile delivery method, there was an increase in DCs’ ability to secrete adjuvant chemicals intercellularly [58]. An imidazoquinoline derivative, acting as a small-molecule TLR7/8 agonist, was covalently bonded to each chain of the polymeric nanogel [58]. At the endosomal pH of nanogel vaccines, adjuvant administration tests have demonstrated a significant increase in effectiveness and safety.

For greater therapeutic effectiveness, it was shown that combining nanogels’ temperature and pH responsiveness boosted the targeting and retention of adjuvants [59]. A dual-responsive system may control the tissue and intracellular delivery of a cancer vaccine.

For a cancer vaccine to be successful, the antigen and adjuvant must be injected concurrently. Several prior investigations [60] have shown this to be true.

**Figure 2:**
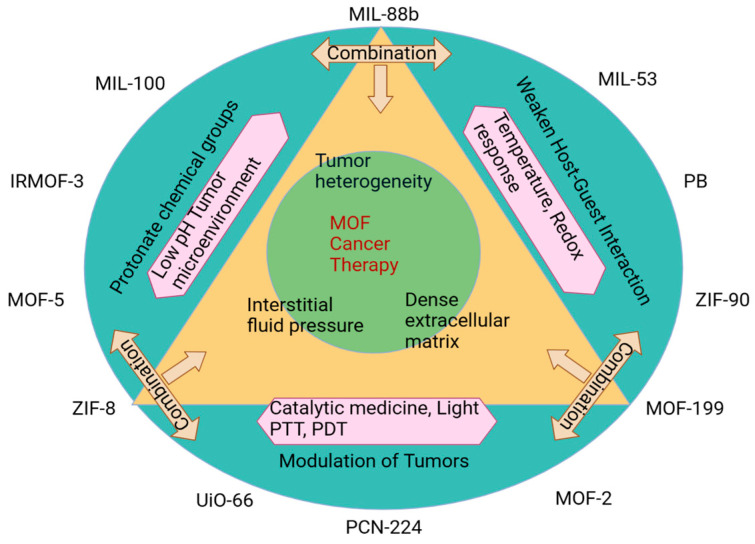
A MOF-based pH-sensitive vaccine delivery method for cancer MIL (Material Institute Lavoisier), IRMOF (Isoreticular Metal–Organic Framework), MOF (Metal Organic Framework), ZIF (Zinc Imidazole Framework), UiO (Universitetet i Oslo), PCN (Porous Coordination Network), PB (Lead), PTT (Photothermal Therapy), and PDT (Photodynamic Therapy).

Using responsive biomaterials, co-delivery has been accomplished. The acid-degradable MOF-based NP was developed by Duan et al. for the delivery of melanoma antigens and adjuvants by coordinating lanthanide ions with GMP (Figure 2) for both encapsulation and surface binding. This MOF-based delivery mechanism was loaded with an ovalbumin (OVA) model antigen, and Watson–Crick base pairing permitted the integration of an adjuvant, a TLR-9 antagonist, in a simple one-pot approach. MOF-sized nanoparticles’ LN and DC internalization strategies were fine-tuned for this aim (with a 30 nm diameter). The B16-OVA mouse model, which expresses the OVA gene, was vaccinated more successfully and efficiently using a formulation that degraded quickly at pH 5.0.

With an acid-labile chemical structure, pH responsiveness may be easily and quickly created. More pH-labile chemical structures, such as the reversible link generated by maleic anhydride and primary amine [61], may increase the efficiency of cross-presentation to the library of sensitive biomaterials. In addition to the most often investigated biomaterials, hydrogels and microneedles may potentially be employed for vaccinations against cancer that respond to changes in pH [62,63].

### 5.2. Acid-Triggered Phase Transition-Based pH-Responsive Biomaterials

Using charged peptides and polycarboxylic acids, which are non-degradable pH-responsive biomaterials, is also widespread in vaccine administration. After protonation, the biomaterials’ increased hydrophobicity causes a considerable phase shift in acidic conditions. Hydrophobic interactions between the phospholipid hydrophobic domain and the protonated polymers may let vaccine components leave the endosome more easily [64].

Stayton and associates looked into several polymers with carboxyl groups for their use in the administration of vaccines. This group’s model antigen poly (propyl acrylic acid) combination greatly increased the proliferation and survival of mice with tumors containing the EG.7-OVA gene [65]. As part of their distribution strategy, they employed amphiphilic block copolymers [32,66]. Due to their simple chemical production, for the administration of pH-sensitive vaccines, a wide variety of biomaterials having the property of acid-triggered phase transition have been produced. There is no assurance that the antigens and adjuvants contained in vaccination carriers will be released even if they undergo a phase shift. A disulfide bond, for example, may be added to this biomaterial to speed up when vaccine components are released from transporters after they enter the cytoplasm of an APC.

Phase transitions caused by pH have been mediated by biomaterials, improving cancer vaccine endosomal disruption and intracellular delivery [67,68]. These components are taken up and generate pore-like membrane structures by acidic endosomes because of their secondary and primary structure interactions. Yuba et al.’s pH-responsive fusogenic polymers [69,70] are examples of liposome-based vaccine delivery methods. pH-responsive liposomes have been used to construct a range of medicinal carriers, including cancer vaccines using synthetic mutagens with a comparable phase transition property [54]. Using a synthetic peptide rich in amino acids GALA (glutamic acid–alanine–leucine–alanine) [71,72], Morishita and colleagues described the surface conjugation of exosomes derived from mouse B16F10 tumors. MHC Class I antigen presentation was greatly improved by GALA-exo compared to that of unmodified exosomes. Liposomes are generally better than nanoparticles when it comes to the hydrophilic virus-specific loading capacity and vaccine cargo release speed (NPs). pH-responsive liposomes may have substantial limits since their lipid membranes are more vulnerable to rupture when injected intravenously with cancer vaccinations. By connecting the lipid bilayer, it is possible to stabilize lipid nanoparticle vaccines without affecting their ability to release antigens [73].

Natural and synthetic vaccine delivery techniques might benefit from the use of fusogenic peptides or polymers. If a vaccine, such as the one generated by Qiu et al., is self-assembled, it is possible to attach a pH-sensitive peptide (pHLIPs) to its surface to facilitate endosomal escape. They have an improved capacity to activate and proliferate antigen-specific T lymphocytes if loaded with an antigen such as NY-ESO-1 (NP-pHLIP) [74,75].

### 5.3. pH-Responsive Biomaterials for “Proton Sponge” Effect

Because of the low pH of endosomes, it is important to utilize polycations as buffers because of the high amine group count. For the delivery of nucleic acid payloads, the “proton sponge effect,” which has been widely investigated and analyzed for its probable applicability, may be used. Methods such as these may help cancer vaccines escape endosomes. Tertiary-amine copolymer-based NPs (UPS) may be activated and deactivated in a very limited pH range (a pH change of 0.25) by Gao and colleagues [76]. Because of its potential to trigger CD8+ T cell responses, UPS NP PC7A was shown to be a promising carrier for cancer vaccination. Antigen carrier PC7A NP is also an adjuvant in this vaccine formulation, which shows STING-dependent DC activation. Administering PC7A as a cancer vaccine in mice prevents a wide range of cancers, including melanoma and colorectal cancers, from forming.

### 5.4. Other pH-Responsive Biomaterials

When it comes to cancer vaccines, the notion of integrating a pH-responsive promoter in addition to the other vaccine components is novel. Liu and colleagues confirmed this point by encasing the ammonium bicarbonate (NH_4_HCO_3_) activator and the vaccination payload in a thin-shelled PLGA NP (poly (lactic-co-glycolic-acid) nanoparticles) [34]. When NH_4_HCO_3_ reacted with protons in endosomes, they shattered the NPs’ outer shell, allowing antigens to escape. The co-encapsulation of pH-responsive promoters and antigen distribution control is used in this method.

An investigation of the possible deleterious effects of intracellular CO_2_ and NH_3_ on cells is needed to understand how these gases disturb endosomes.

A considerable amount of work has gone into developing pH-sensitive biomaterials with a wide range of chemical structures for the delivery of responsive cancer vaccines. These biomaterials must be very sensitive to pH changes to fall into this specific category (from 7 to 5).

UPS NPs, which are very pH-sensitive, may promote anticancer effectiveness, as seen by an increased CD8+ T cell response. Improved pH-responsive biomaterial sensitivity is required for more effective cancer vaccinations, and this can only be accomplished by more accurately controlling antigen distribution. An efficient and reproducible preparation method is essential in the medicinal use of biomaterials. Studies in the therapeutic setting are more likely to focus on large-scale materials that have a reliable preparation procedure.

## 6. Redox-Responsive Vaccine Delivery System

There is no doubt that glutathione (GSH) production has long been recognized as having an important role in the rise in the reducibility of the internal environment.

Glutathione reductase often repeats this reductive short peptide to maintain low levels of ROS in eukaryotic cells [77].

**Figure 3:**
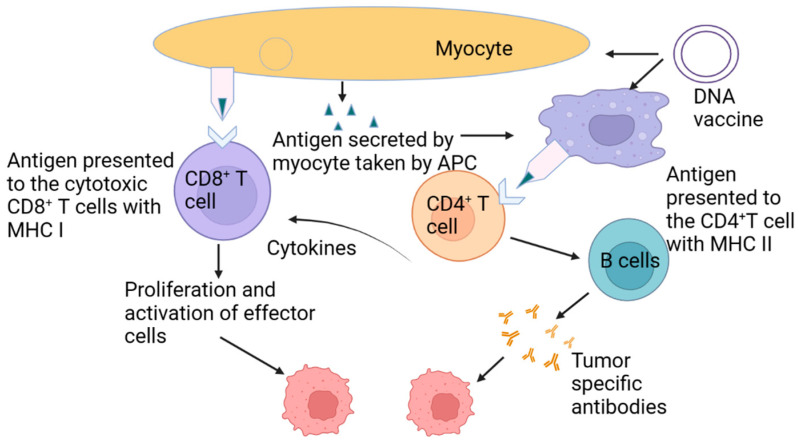
Cancer vaccines using redox-cleavable linkers: a comparison study.

The cytoplasm’s reducibility, which is equivalent to the endosome’s low pH, allows for the intracellular transport of a wide range of cargoes. Intracellular GSH (glutathione) may break a disulfide bond in vaccine delivery methods that are sensitive to changes in redox conditions. Side chains of macromolecules or synthesized NPs may be linked through disulfide bonds to antigenic chemicals. The Hubbell and Swartz groups developed a cancer vaccine delivery method using a redox-responsive delivery mechanism [37,78]. They used a reducible disulfide bond to connect OVA to a polymeric NP. Non-reducing or soluble antigen vaccinations did not produce a robust enough CD8+ T cell response. MHC Class I loading is enhanced, and the escaped endosomes are responsible for this effect, as shown in Figure 3.

Among these redox-sensitive cancer vaccine delivery methods are polymeric NPs, system actions MOFs, and nanogels incorporating reducible disulfide bonds [79,80]. Kramer et al. devised an antigen–adjuvant combination with varied sensitivities to precisely regulate antigen release by taking advantage of a large differential in reduction capabilities within and outside DCs [39]. Both linkers had distinct redox sensitivities; hence, the conjugates were created. The HYN-SS linker is more difficult to cleave intracellularly, whereas the SS linker may be easily cleaved even in the presence of a small drop in activity (i.e., extracellularly). It was a linker that could not be reduced at the time (HYN). Immunized mice were better protected against tumor challenge by HYN-SS linker-prepared prodrugs than by SS or HYN-linked ones. CD8+ T cells’ response to malignancy was amplified by manipulating antigen transit and fate in the cytoplasm, as shown in this work. Wang et al. used disulfide linkage to create a “minimalist” nanovaccine by directly producing remarkable outcomes from the OVA proteins (with decreased free thiols) (mNV).

Cross-linking with OVA was made easier by using a CpG with a free thiol group. This form of vaccination was almost 100 percent successful for CD8+ T cell proliferation in vivo without the need for extra carrier materials. As a consequence of MNV vaccination, C57BL/6 mice may be able to effectively suppress and prevent the development of B16-OVA melanoma. Toxic- and immunological-response-free mNV enhanced antigen and adjuvant loading capacity while decreasing carrier toxicity [80].

Redox-responsive cancer vaccines have a smaller chemical design than pH-responsive biomaterials because of their disulfide composition. Disulfide bond cleavage may be hindered by the cell’s acidic environment. More redox-sensitive and responsive cancer vaccine delivery polymers are urgently required. The combination of sensitivity and redox responsiveness may lead to more efficient endosome egress and faster antigen release from the cytosol, and this may be a feasible method in the future cellular immune response against cancer.

## 7. Light-Responsive Vaccine Delivery System

**Figure 4:**
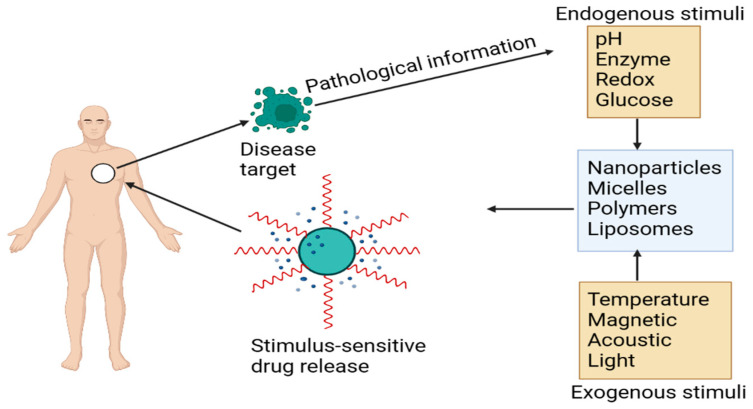
PheoA and PEI-based light-activated vaccine delivery system.

Stimuli-responsive vaccine delivery systems combine external triggers such as light and radiation with internal triggers such as those already mentioned to achieve a precise spatiotemporal management of antigen release. To manufacture light-activated vaccines, photodynamic therapy and vaccine components are often mixed in a carrier. ROS may lead to the release of antigens if a certain wavelength of light is utilized to activate the photosensitizer, breaking the endosome membrane. “Photochemical internalization” (PCI) is the term used to describe this process. TPCS2a, a photosensitizer, is being used by Hkerud et al. to create light-responsive vaccine delivery systems, including liposomes loaded with antigens and soluble antigen complexes [81]. The CD8+ T cell response in mice exposed to 435 nm visible light was much greater than in mice not exposed to irradiation. Using PCI to prevent antigen molecules from exiting endosomes and cross-priming CD8+ T cells is a key part of this study’s results. A light-responsive vaccine delivery method based on PEI and Pheophorbide A (PheoA) was developed by Zhang et al. (Figure 4). PheoA-PEI/OVA NPs were created by electrostatically coupling the self-assembled PheoA-PEI to the model antigen OVA in an aqueous solution. To tear down the endosomal membranes of DC2.4 cells, we employed light at a wavelength of 670 nm. The researcher used the transdermal vaccination of DC2.4 cells surrounding the E.G7-OVA tumor, PheoA-PEI/OVA NP injection, and proper wavelength light irradiation before injecting the animals to demonstrate by CLSM that the antigen was released and supplied intracellularly after being activated by light. When compared to non-responsive NPs or free OVA, light-responsive NPs were more effective in preventing tumor formation. Photosensitizers and pH-responsive promoters have a similar function when a cancer vaccination is delivered through the endosome. Photosensitizer-based vaccination carriers will require more research. As a result of the biologically transparent window, NIR light penetrates further into skin or mucous membranes than lower-wavelength light. Antigen molecules and hyaluronic acid were loaded into gold nanoparticles (HA-OVAAuNPs) in recent work by Cao et al. (HA). Nanovaccines were absorbed by APCs that have surface-bound HA and CD44 receptors. NIR radiation was converted into thermal energy using AuNP [82]. NIR laser irradiation for three minutes resulted in significant local temperature increases of up to 42.3 °C in BMDCs treated with HA-OVA-AuNP. Antigen molecules cannot enter CD8+ T cells’ cytoplasm unless the endosome barrier is disrupted at a high temperature. The proliferation of CD8+ T cells increased, leading to a substantial reduction in tumor development in mice with an EG.7-OVA tumor.

Systems relying on internal triggers, such as spatial control, have a disadvantage since they are less focused on the activity of the released vaccine components in the targeted tissues. Externally triggered vaccine delivery methods have this advantage. Biomaterials that may be used to administer cancer vaccines can be developed with the help of a huge library of photosensitizers that are already available [83]. Further in vitro and in vivo investigations are required to demonstrate the therapeutic potential of cancer vaccines that respond to light and are now in the research and development stage. NIR-light-sensitive biomaterials, as opposed to UV or visible light-responsive biomaterials, have a higher chance of success in therapeutic applications because they penetrate deeper into tissues.

## 8. Molecular Recognition-Responsive Materials

Designing bio-materials that can release therapeutic cargo in response to the binding of a specific molecule or molecules is a relatively recent breakthrough in the field of smart materials. All of these materials that respond to molecular recognition use competitive binding to a target to trigger the release of cargo under specific conditions. Because of their high specificity, these materials can be delivered precisely to the locations where sufficient quantities of the analyte are present. Higher concentrations of characteristic surface receptors or signaling proteins are typically found at drug release target sites. Scientists are employing a wide range of methods to develop therapeutic materials that can release drugs in response to interaction with disease-associated molecules that are particular to a given area. A strong affinity for an antigen or ligand template molecule can be engineered into a molecularly imprinted polymer (MIP) formulation. The template molecule is combined with free monomers and cross-linking agents to produce the desired substance. Following polymerization, the template molecules can be removed by washing, leaving behind a recognition scaffold with high affinities for the target molecules. When bound to their respective template molecules, these MIP hydrogels are able to undergo a variety of physical transformations. For instance, lectin and antibody molecules were used to imprint a hydrogel that contracts in response to the presence of the tumor-specific biomarker glycoprotein fetoprotein (AFP) [84].

## 9. Magnetic Responsive Materials

In addition to electromagnetic fields, various external triggering and targeting stimuli have been used in drug delivery methods. Therapeutic applications of programmable magnetic NP systems rely on either external magnetic guidance or local drug activation via induction heating with the alternating magnetic field. Furthermore, magnetic resonance imaging (MRI), which uses a magnetic field to image anatomical or physiological processes in the body, can be utilized to identify magnetite NPs (MNPs) non-invasively. Consequently, there is a great need for MNP formulations that can perform multiple functions at once, such as magnetic drug delivery, MRI diagnosis, and thermosensitive chemo-therapy, to improve cancer therapies [85].

## 10. Other Responsive Vaccine Delivery Systems

APC absorption and vaccine distribution in the cytosol are now more responsive to cancer vaccine delivery strategies than previously thought. The fusion of liposome vaccinations with APC membranes may promote the cross-presentation of antigens. The researcher showed that ultrasound may be used to stimulate APC–APC fusion using a PEGylated bubble lipoplex with lactose linked to the surface of a DNA vaccination [46,47]. To improve the effectiveness of gene transfer, the so-called “sonoporation approach” has been frequently used. A liposomal vaccine was able to stick to APCs because they had many mannose receptors on their surfaces. Antigen-encoding DNA (pDNA) was transported straight to the cytoplasm of the APC without the need for endocytosis thanks to the bubble lipoplex’s ultrasonic fusion with the cell membrane. Ultrasound-aided CD8+ T cell activation and cytokine production in a B16F10 melanoma mouse model lowered tumor formation and increased survival. Until now, the use of physical triggers to regulate vaccination distribution has been limited. Ultrasonography-sensitive cancer vaccines have a promising future.

## 11. Conclusions

In the development of cancer vaccines, biomaterials capable of generating strong cellular immune responses specific to antigens have shown tremendous promise. Cancer vaccine dispersion has so far been unable to be controlled by biomaterials that respond to stimuli, despite this being the primary goal of most current research. A pH-sensitive biomaterial is the most common responsive biomaterial for cancer vaccine distribution to date. Endosome disruption mechanisms and pH-sensitive chemical moieties are among the many tools they use. Due to the high levels of oxidative stress in the cytosol and the capacity to use light as an external trigger, redox and photo-responsive materials have been underutilized in the administration of cancer vaccines.

Cancer vaccine delivery employing responsive biomaterials that utilize triggers other than enzymes, electricity, and mechanical force has so far not been studied in any depth. For stimuli-responsive delivery, enzymes are an excellent trigger [86,87] because of their great efficacy and specificity. For this purpose, enzymes have been extensively studied. Enzyme-responsive biomaterials, such as liposomes and polyester nanoparticles, provide a novel route to study the breakdown of cancer vaccine carriers [88]. This review paper aims to shed light on how stimuli-responsive biomaterials aided in dispersing the cancer vaccine. More stimuli-responsive biomaterials can move from the lab bench to clinical trials with the ultimate goal of reducing patient suffering by formulation as a result of future research goals and continual innovation.

## Figures and Tables

**Table 1:** Developments in the use of stimuli-responsive biomaterials for cancer vaccines.

Stimuli	Responsive Chemical Structure	Delivery System
Temperature-responsive biomaterials for improved LNa targeting	NIPAM	Immunization–polymer conjugate
Biomaterials that are responsive for improved cross-presentation Acidic surrounding (pH)	Acetal bond Coordination bond Carboxyl group NH_4_HCO_3_ Tertiary amine Fusogenic peptide	Cross-linked polymeric NPc MOFd
Reductive environment (redox)	Disulfide bond	NP polymer Conjugated vaccines Environmental reduction (redox) Sulfide bond MOF Adjuvant/Antigen NP
Light (Visf or NIRg)	TPCS2a PheoAi Gold NP	Conjugated antigen–polymer Inorganic NP polymer Polymeric nanoparticle
Ultrasound	Bubble lipoplex	Liposome

## Data Availability

Data sharing not applicable.
